# Key to Cholesterol's Role in Nematode Development

**DOI:** 10.1371/journal.pbio.0020345

**Published:** 2004-09-21

Cholesterol has a bad rap for its association with human heart disease. But actually cholesterol and other sterols are essential for a wide variety of organisms. For most eukaryotes—organisms whose cells have nuclei—sterols reside in the cell membrane and play major structural roles. Sterols keep cell membranes flexible, for example. These chemicals also hinder leakage of ions across the membrane, which is crucial in order for muscles to contract and nerves to conduct signals.

For the tiny (eukaryote) nematode worm Caenorhabditis elegans, sterols are a dietary staple. Worms can't make these chemicals from scratch, just as humans can't make vitamin C or the essential amino acids, so they have to harvest these chemicals from their surroundings. If nematodes hit hard times—they can't find enough sterols, say, or are starved or overcrowded—they can delay developing into adults. Instead, they enter a stage called a dauer in which they don't eat and hardly move a muscle. In this state, they can persist several months—many times their normal lifespan—and then revive when conditions improve.[Fig pbio-0020345-g001]

**Figure pbio-0020345-g001:**
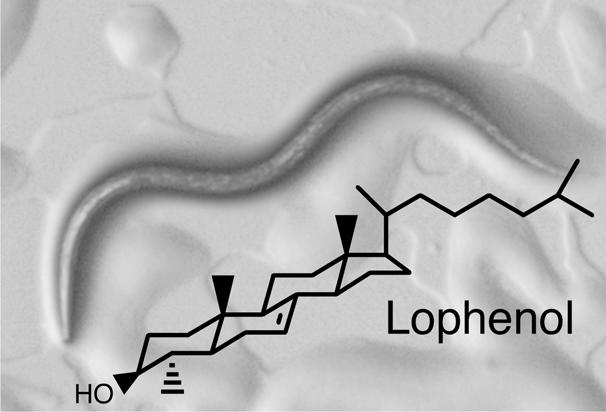


Though C. elegans is extensively studied, there's still controversy over the role of cholesterol in this organism. To develop into adults, the nematodes need only small amounts of cholesterol in their diet, suggesting cholesterol does not play a major role in their membranes. Instead, nematodes—like many other eukaryotes—might use cholesterol to make hormones, which are typically active at very low concentrations. Such hormones could play a key role in the worms' development into either adults or dormant dauers. But no one had found any nematode hormones derived from cholesterol—until now.

In this issue of *PLoS Biology,* Teymuras Kurzchalia and colleagues show definitively that cholesterol does not play an essential structural role in C. elegans. Rather, cholesterol is the precursor for a hormone—or set of hormones—key in the worms' development into adulthood and thus key for reproduction. The researchers have partially purified this cholesterol derivative and named it gamravali, from the Georgian word for reproduction, “gamravleba.”

When on sterol-free diets, all larvae showed arrested development, becoming dormant dauers. But, surprisingly, the concentration of cholesterol they needed to develop into adults was miniscule, around 20 nanomoles. When given scant amounts of cholesterol, the worms converted some of it to a sterol called lophenol. The researchers found, however, that supplementing a sterol-free diet with lophenol was not enough to sustain development into adulthood. Apparently the worms need cholesterol, which is fed into two distinct pathways: one makes lophenol and another makes the hormone gamravali.

The researchers have only partially purified gamravali, so they don't yet know its molecular weight or composition or even whether it is a single molecule. But by working with mutant worms, they have begun to pin down where gamravali acts in the worms' developmental pathway. One mutant C. elegans line, for instance, was unfazed by the cholesterol-free diet. These mutants were missing the *daf-12* gene, one of a set of genes crucial in nematode development and aging. On the cholesterol-free, lophenol-supplemented diet, these mutants developed into normal adults. Other mutant lines that each lacked one of several other *daf* genes, however, developed into dauers when deprived of cholesterol. In this way the researchers found where gamravali acts in the worms' developmental pathway: the hormone gamravali likely comes into play before *daf-12,* but after the other *daf* genes. Kurzchalia and colleagues are currently working to further purify gamravali and identify exactly how it gives cholesterol such a crucial role in the worms' lifecycle.

